# Hospital and Institutional News

**Published:** 1916-01-22

**Authors:** 


### HOSPITAL AND INSTITUTIONAL NEWS.

### THE BOARD ROOM ATMOSPHERE.

A minor field of hospital research, which might yield fruitful results to anyone with sufficient leisure and resource to explore it, is that provided by patients' letters. An analysis of these, drawn from a sufficiently wide area, would be amusing and instructive, as showing the often unexpected things in and about a hospital and its routine which have favourably impressed the patients. We say favourably, not because a favourable impression is absolutely universal, but because, as a rule, it is gratitude rather than criticism which inspires these epistles. The patient, who is also a critic, is generally too timid to go beyond an anonymous letter in the local newspaper; and anonymous letters are no good. The reputation of a hospital largely depends on the porter and the nurse, who have it in their power to create the impression of kindness, or the reverse, which the patient will afterwards carry away, and pass on to his friends and neighbours. But, as a recent letter of thanks to the Poplar Hospital shows, a board meeting also has a power of exciting affectionate respect. Thus, one patient lately wrote to Mr. J. G. Broodbank, the acting chairman, a letter containing these words: "I have the pleasure," he said, "of enclosing one guinea cheque. I was much impressed with the meeting last Wednesday, everything seemed so human." As we have pointed out for twenty years, it is not this fact or that about a hospital by which its worth is to be judged; neither equipment, nor "efficiency," nor size, nor even management. The thing alone by which the standard of a hospital can be measured, which points unerringly to the measure of its worth, is its atmosphere. For the test of a hospital is whether it is a place in which a sick person would desire and do well to be. Every patient, in some degree, realises the meaning of atmosphere. The above letter, therefore, pays a high compliment, for hospital and its board react on one another, and a committee which strikes the uninitiated as humane is one which has probably got men, and not mere managers upon it.

### THE SATURDAY FUND'S FIGURES.

The fact that the income of the Hospital Saturday Fund, on its General Fund Account, has gone up by some £6,500, and reached a total of £46,738—as compared with £40,676 a year ago—is very remarkable, and coming, as it were, like a New Year's present to the hospitals, is most encouraging to all who have the voluntary system near at heart. We have only to recall the fact that the Metropolitan Hospital Sunday Fund's accounts also show an increase of over £10,000 on the income for 1914 to recall also the healthy and progressive condition of the King's Fund, and we realise at once that the war has brought out the best in the voluntary system, and that its foundations are stronger than ever. When we ask ourselves what are the causes which have led to this increased generosity, many factors have to be considered. Gratitude to the hospitals for their work for the wounded; the fact that the war has induced many new persons to subscribe who have never previously done so: both these account for much. For it must be remembered that the number of people in the past who have been subscribers to any form of charity has been relatively small. The great difficulty always is to induce new people to give, and the success of many appeals in the past has been secured by the generous sacrifices of habitual or well-tried donors. But no one has escaped the appeal of the war. In one way or another it has touched us all. Hence, in part, the success of the great Funds. But even so, so far as the Saturday Fund is concerned, the new total was nearly reached by the record established in 1911. Legacies or other windfalls have to be considered in this connection, and we note that £11,406 was received at the head office of the Saturday Fund within the last fortnight of the financial year. No doubt Mr. A. W. Davis could throw an interesting light on this. We can only hope that these figures will indicate that the new impetus is a permanent one, and that the Saturday Fund will progress towards an income of £50,000 a year without any going back.

### A PATIENT'S GRATITUDE AFTER FIFTEEN YEARS.

In August 1912 a working man appeared at the front entrance door of Addenbrooke's Hospital. The Secretary-Superintendent seeing the man there, asked him if he had been attended to, and he replied in the negative. He said he had cycled a long way that day to bring some money to the hospital. The Secretary-Superintendent told the man he would gladly receive the money, when he put ten pounds in gold into his hand and proceeded to go. The Secretary explained that he must issue an official receipt, but the welcome visitor refused to give either name or address, and only after considerable persuasion did he state that the donation was as a slight acknowledgment of the treatment which he had received in the Griffiths Ward (then medical) twelve years before. Quite recently a visitor, having every appearance of a farmer, walked into the Secretary-Superintendent's office at Addenbrooke's Hospital and handed to the Secretary ten pounds in Treasury notes. When asked his name and address the visitor flatly refused to give either. The Secretary remarked that the incident reminded him of a poor working man who had handed him a similar amount, under identical circumstances, about three years ago. The visitor replied, "I am the same man, but am a little better off, and I gladly give this ten pounds as an expression of gratitude for the great kindness I received as an in-patient fifteen years ago. I hope to bring a further contribution at some future time." What an example to those who receive benefits in our voluntary hospitals throughout the world! May all old patients who are able to follow it come quickly with the same modesty to their hospital!

### THE DISREGISTERED MAN AND THE WAR.

One of the weekly papers, which is more distinguished for fertility in invention than for patience in investigation, has propounded a truly marvellous scheme for augmenting the cadres of the Royal Army Medical Corps. This is nothing less than the restoration to the Medical Register of all those struck off it for various acts of misconduct, a proposal which is ingenuously described as giving the disregistered a "second chance." The rabble who would thus be readmitted into the profession are described as "outcasts, whose only fault was some breach of etiquette"; a most picturesque phrase, doubtless, but a hopeless perversion of the facts. For the benefit of those who may cherish the belief that medical men are often struck off the register for purely technical crimes of this kind, it may be well to state that the great majority of such "outcasts" are disregistered for offences such as drunkenness, fraud, theft, and immorality. In the very few cases where misconduct in a solely professional sense is the offence proved, not only second but third and fourth chances are always given before, not after, the culprit is struck off; and the misconduct has to be very gross before the offender is even cited before the tribunal of the General Medical Council. We do not think the Royal Army Medical Corps will be very grateful for the suggestion that it should be asked to receive the submerged tenth of the medical profession.

### AN ENDOWED WARD AT CARDIFF.

In another of these Notes we draw attention to the gift of a bundle of securities to a Scotch institution which will bring in an income of between £300 and £400 per annum. In Wales a rival to this is seen in the endowment of the seventh of a series of beds during the past twelve months, with the result, of course, of a similar sum being annually secured to the Southern institution, King Edward VII. Hospital, Cardiff. When it is added that four of these beds have been endowed within a month, it shows that this policy, popular at Cardiff, is bearing good fruit. The gift of securities noted elsewhere was the result of a special appeal and, in that sense, a piece of good fortune; the policy of endowing beds is one that can only be practised on a large scale by institutions in great industrial centres where rich men, or wealthy companies, command resources which admit of such handsome gifts. There is, perhaps, more room for latitude in the treatment of gifts for the endowment of beds than in the gift of a sum earmarked for investment; but all such endowments should be placed to the credit of the capital account, so that the bed thus "endowed" really represents an accretion to the invested funds of the institution.

### POOR-LAW RELIEF.

In common with other Unions the recently published figures of Poor-Law relief in Leeds for the year 1915 show a marked reduction on previous years. The recipients of outdoor relief have diminished by about 12 per cent., and the poor in institutions by nearly 20 per cent. Unfortunately this reduction in numbers is not accompanied by a similar decrease in expenditure. The increase in the cost of living has just about balanced the saving due to the fewer numbers. Similar results are noticeable in practically all the returns from the Poor-Law Unions. The reduction in the number of the inmates of Poor-Law institutions has been of great value to the country in that it has enabled Boards of Guardians to place a large number of their institutions at the disposal of the War Office. There still remain, however, a large number of workhouses and Poor-Law infirmaries which are half-empty. A great economy not only in money but in labour would be effected by closing many of these institutions and distributing their inmates amongst neighbouring Poor-Law buildings. It may be that the present reduction in the numbers of the indoor poor will not be permanent, and that some of the institutions so closed might have to be reopened after the war, but that is no argument against effecting an immediate saving.

### SYMPATHY AND CONGRATULATIONS IN WAR-TIME.

The committee of a voluntary hospital in its ordinary course has much detailed and some interesting work. Since the war began its work has increased, and the interest of it has quickened owing to the many new points and difficulties which have arisen from time to time. After eighteen months of war the work before such a committee is beginning to offer new opportunities for sympathy and congratulation, which, when availed of, cannot fail to help the hospital by knitting closer the ties of co-operation and kindly feeling between the committee, the governors and subscribers, and all workers for the sick within its jurisdiction. At the monthly meeting of the board of the Leicester Royal Infirmary, just held, incidents of the war were prominently under notice. The board had the privilege and pleasure of noting the award of the Royal Red Cross (First Class) for valuable service rendered in connection with the war to Miss C. E. Vincent, the matron, who has also been discharging the duties of principal matron of the 5th Northern General Hospital. His Majesty has also been pleased to award the Military Cross to the following past and present members of the medical staff: Captain Claude Gordon Douglas, M.D., R.A.M.C., (temp.), son of Lieut.-Colonel Claude Douglas, F.R.C.S., honorary consulting surgeon to the infirmary; Captain W. H. Barton, M.R.C.S., L.R.C.P., R.A.M.C., who at the outbreak of the war was a resident medical officer at the Leicester Royal Infirmary; and to Captain W. S. Fielding Johnson, second son of Mr. T. Fielding Johnson, junior, J.P., chairman of the house committee, who was also recently promoted in the Royal Flying Corps. Leicester Royal Infirmary thus shares in the Record List of honours recently published, to the extent of one Royal Red Cross (First Class) and three Military Crosses, a notable circumstance which called for a congratulatory resolution of the board in each case. Very many voluntary hospitals up and down the country have been similarly honoured through their workers on war duty. We shall welcome particulars from every one of them if the committee's representative will forward them in due course. There was, too, a note of sorrow at the Leicester meeting, for the board directed an expression of deep condolence to be forwarded to Dr. Hooper, the father of Staff-Surgeon A. O. Hooper, R.N., M.B., B.Ch., a former resident medical officer, who lost his life in the sinking of H.M.S. *Natal.*

### ROBBING THE HOSPITALS.

A letter on this subject from Viscount Knutsford will be found in our correspondence column. It insists upon the utility, in the interests of all those responsible for the administration of our hospitals and charities throughout the Empire, of placing The Hospital in a position, whenever a robbery takes place, "to publish how the swindler worked his swindle." In the past, for various reasons, there has been evidence of a desire not to publish information on matters of this kind, which has caused offenders probably to regard their offences as of relatively small importance, for the consequences to themselves might be relatively slight owing to the suppression of the facts. So tenderness further encourages rather than defeats the temptations to employees, who may feel, as offenders have felt, that if discovered the consequences may be ignored. A further important point is made, that if it were the rule of all charitable institutions to supply the facts in each case for publication in The Hospital, a record would be available of the nature of every institutional swindle or scheme of theft. Detection and prevention, in consequence, must be rendered much more certain than they can ever be so long as details of crimes of this class are suppressed. We shall be glad to open our columns to correspondence on this subject, but we are gratefully conscious that swindles of the type referred to are relatively rare.

### A MATTER FOR REGRET.

We are sorry to notice an article in our contemporary, the *Hospital Gazette*, which is the organ mainly of the junior lay officers of hospitals. It constitutes a veiled attack on the Uniform System of Hospital Accounts, arising on a paper read by Mr. Lionel Lemon, F.C.A., honorary auditor to the Incorporated Association of Hospital Officers. Mr. Lemon's point appears to be that the balance-sheet adopted in connection with the Uniform System of Accounts by the three great metropolitan distributing funds is too good, *i.e.* elaborate, and ought to be abolished in favour of something much simpler which an ordinary man who is not an accountant can readily understand. Mr. Lemon appears to have fallen into error on another point by his statement that a hospital converts itself into a company under the Companies Acts 1862 onwards "for the purposes of registration under the Friendly Societies Acts." We should be glad to know the grounds and authority on which this statement is made, for we believe it to be without foundation from the hospital's point of view, and that there is no legislative or other enactment which compels a hospital so incorporated to be registered for the purposes of the Friendly Societies Acts. We may add on the first point that no young worker who wishes to train himself for high office in a large voluntary hospital can hope to succeed unless his standard of work, life, and conduct is of the highest. Success in hospital work rests largely upon an infinite capacity for taking pains, a principle which every experienced hospital administrator, as the outcome of his knowledge and experience, upholds and enforces.

### THE DILEMMA AT BRADFORD.

The proposals for extending the War Hospital at Bradford have caused much heart-searching in the city, and as a decision is to be expected this week it may be well to review the position. The Bradford Board of Guardians have already placed a large part of their premises at Horton Lane at the disposal of the military authorities for the accommodation of wounded soldiers, but the result of a further request for more beds from the Army Council is a proposal to hand over the entire buildings at Horton Lane, except the casual wards and the medical officer's house, to them. While the buildings, it was proposed, would be under the Guardians' control, the administration would be in the hands of the Commanding Officer. The scheme, however, was strongly contested at a Guardians' meeting a fortnight ago, on the grounds that more time was required for its consideration, so that the present inmates might be suitably housed elsewhere; that the Guardians should retain control of their own establishments; and that the cost of providing temporary buildings for those at present in St. Luke's Hospital and the Union House would be far in excess of the £25,000 estimated. The chairman, Mr. R. S. Dawson, however, urged that everything should be done to provide 1,400 beds, and that the military, once in charge of the whole of the buildings, should be left to administer them in their own fashion. At present the institution is jointly managed. There is properly a strong feeling also, at which the Chairman hinted, that any attempt to thwart the scheme would be considered as lacking in patriotism.

### EMPLOYMENT FORBIDDEN TO PARTIAL CONVALESCENTS.

Of all the rules of friendly societies, that is the most rigid and unalterable which precludes a man "on the club" from doing any sort of work or trying to earn any money. Not a few criticisms have been levelled at this rule, and with the present shortage of labour this point takes on a new and greater importance than before. From the medical point of view there is everything to be said for a relaxation of this rule in the cases of certain convalescents and "chronics." After nearly fifty years' experience, Dr. John D. Lloyd, the M.O.H. for Chirk Rural District, is convinced that this rule has been the undoing of many a man. He strongly urges that such cases should be allowed to earn what they can at any form of labour, and that the club or society should make up the deficit until the individual is certified as fit for his customary work. At the present time farmers would probably be glad to give employment to some of their old labourers who are now "on the club," and thus prohibited from availing themselves of the offer. The obvious reply to this suggestion by the secretaries of friendly societies is that it will be so difficult "to draw the line." But in the near future we shall all have a good many new lines to draw to tide us over the consequences of the war, and it should not be beyond the administrative capabilities of society officials to find means to allow members on the funds to help themselves back to health by doing part-time work.

### NO MORE GERMAN MEASLES.

German measles has again sprung into prominence as the result of its inclusion in the new Regulations requiring it to be notified as well as measles. Attention is thus once more directed to the unsuitability of this name for a disease which has nothing whatever to do with measles, and does not protect against the major complaint, officially known as morbilli. Having regard to the fact that German measles is such a benign disease the qualifying epithet is singularly inappropriate. The universally disseminated anti-Teutonic spirit of the times should facilitate the dropping of this unfortunate synonym for the disease now commonly known in medical circles as rubella. Unluckily, official sanction has been accorded to the term German measles as the popular alternative. There is, however, another recognised synonym which could well replace it in vulgar parlance, that is "rose-rash," for this or "epidemic rose-rash" is mentioned in all text-books of medicine as an alternative title to this infectious disease. Not many years ago the German name, "rötheln," was more commonly applied to it than the word "rubella." Having practically got rid of one foreign title, is it not possible to graft permanently on to the popular nomenclature the simpler name, in every way apposite, of rose-rash?

### AN INQUEST ON A DEATH FROM MEASLES.

An inquiry held by the city Coroner of Nottingham on January 4 into a death from measles must be, if not the first, one of the earliest inquiries of this nature since the coming into operation of the new regulations regarding the notification of measles. The circumstances of this particular case are commonplace and unfortunately only too typical. A child of fifteen months died on the fourth or fifth day of an attack of measles without having been attended by a medical practitioner. Another child in the house also had the disease, and apparently the cases were not notified, both parents expressing ignorance of the new regulations. A knowledge of this fresh responsibility is bound to be slow in filtering down to working-class homes, and the idea that measles is a disease for which it is worth while calling in a doctor will take time to permeate this stratum of society and higher ones too. The excellent work of our district nurses may be counted upon as a very important factor in securing benefit from these regulations. It is interesting to note that to their verdict at the above inquest the jury added a rider to the effect that "there was a certain amount of neglect on the part of the parents, but they did not think it was any more than carelessness." Obviously nothing more severe could justly be said at this early stage of the working of the measles regulations.

### UNPATRIOTIC COUNCILLORS AT YORK.

Considerable difference of opinion was expressed at a meeting of the York City Council on the question whether the tuberculosis officer, Dr. J. Bell Ferguson, should receive permission to accept a commission in a Sanitary Corps of the R.A.M.C. The Health Committee reported in favour of allowing him to do so; but the upshot of the discussion between sundry aldermen and councillors was that the amendment refusing permission to Dr. Ferguson was carried by a large majority. The speeches at the meeting make an interesting commentary on the curious attitude of some public men to the war on the one hand, and the tuberculosis campaign on the other. The work of the tuberculosis officer in York is obviously important, but no one, least of all Dr. Ferguson himself, would attach such an exaggerated influence on the death-rate from phthisis to the work of this tuberculosis officer as some of the speakers seem to have done. A study of the epidemiology of consumption forbids it. On the other side, how can their views on the seriousness of our position in the war be reconciled with the action of the York City Council in trying to prevent a medical man, with the special sanitary qualification possessed by Dr. Ferguson, from assisting in preserving the health of our troops? Judging from the date of his degree, this tuberculosis officer is presumably a young man, and it is well known that the sanitary sections of the R.A.M.C. are in great need of officers with special experience. It is ridiculous to imagine that the incidence of tuberculosis in York will be appreciably altered by the absence of a tuberculosis officer; and even if it were, what is the value of civilian lives, at such a time as the present, as compared with those of our fighting men?

### COMPULSORY POWERS TO BE APPLIED TO CONSUMPTIVES.

The idea that the principle of compulsory isolation should be applied to certain cases of pulmonary tuberculosis is not now regarded as outside the sphere of practical sanitary administration. The matter is, indeed, one that is beginning to be freely discussed. At Chester recently a conference was held at which it was suggested that compulsory powers should be acquired in regard to the isolation of persons suffering from infectious forms of consumption and also power to prevent patients leaving residential institutions against the advice of the doctor responsible for their treatment. More recently still the County Insurance Committee at Denbigh discussed at length a report of the Sanatorium Benefit Sub-Committee, in which the findings of the above conference were referred to. It was agreed that the campaign against tuberculosis was seriously hampered by the fact that local authorities were without power to treat dangerous tuberculous patients in the same way as persons suffering from other infectious diseases. On the motion of Mr. W. R. Evans, it was decided that a copy of the resolutions passed by the conference should be forwarded to all sanitary authorities in the county, with the hope that attention would be called to the questions raised. In the area of yet another local authority compulsory isolation has within the last few days actually been put in force in one instance.

### A LIGHT ON THE CAUSATION OF PELLAGRA.

An important advance in our knowledge of that mysterious disease, pellagra, has been made as the result of an experiment in dietetics described in the United States Public Health Reports for last November. Eleven white men were fed on a restricted one-sided diet, consisting mainly of cereals (carbohydrates), with cabbage, potatoes, sugar, and coffee, but without vegetable fats. Another group of men was carefully observed as a control. Both sets of men were inmates of an institution and lived in the same hygienic surroundings and did the same work, but the controls received the ordinary mixed diet of the institution. In this place no case of pellagra had been previously recognised, but in the course of five months six of the eleven men developed signs of this disease and the diagnosis was confirmed by experts. An interesting observation was made during this "experiment" on the site of the first appearance of the dermatitis in these patients, in each case it was noticed first on the scrotum. Had it not been for the thoroughness with which these men were daily examined all over, it is probable that this fact would have escaped record.

### THE ENDOWMENT OF ABERDEEN ROYAL INFIRMARY.

A very gratifying response to the special appeal which has been lately issued on behalf of the Royal Infirmary, Aberdeen, is the generous gift by Sir Alexander McRobert of securities sufficient to yield the institution an income of £373 a year. This was a second thought which took the place of a promise to give the last £400 necessary to make up the deficit. It is needless to point out the value of such gifts, which come more usually under the heading of legacies than of donations. Sir Alexander McRobert, who was knighted in 1910, is the director of the Cawnpore Woollen Mills Company. A native of Aberdeen, he went in 1884, at the age of thirty, to Cawnpore, where he prospered and took some part in Indian public life. It may be added that the above gift is also a proof of Mr. Alexander Dupper's persuasive powers, since as chairman of the board of management he had an opportunity of explaining the true bearings of the hospital's position to Sir Alexander at a personal interview.

### AN ENDOWMENT FOR RESEARCH.

There is not yet anywhere near sufficient endowment for research in Great Britain, especially in England; it is good news, therefore, that a generous benefaction has just augmented the opportunities that already exist. It is from Scotland, at Aberdeen University, that this is reported. England could do with benefactors as enlightened and public-spirited as Sir Alexander McRobert, whose gift to the University will provide an endowment of £750 a year for a Georgina McRobert lectureship on pathology, with special reference to malignant diseases. The same gentleman, as noted in the preceding paragraph, has just provided the Aberdeen Royal Infirmary with an endowment of £373 a year. Before he went to India Sir Alexander no doubt learned to appreciate the value (and the underpayment, probably) of scientific studies as Neil Arnott lecturer in experimental physics at the Aberdeen Mechanics' Institution, and as lecturer in chemistry at Robert Gordon's College, Aberdeen.

### AN IMPORTANT DISTINCTION.

The official spokesman of the War Office in the House of Commons (Mr. Tennant) has apparently not yet adopted the nomenclature of enteric disease which has commended itself to every authority who has written on the subject since the war began. He still uses the term enteric fever as synonymous with typhoid fever, and excludes the paratyphoid fevers from the category of enteric fever. Hence much quite needless confusion has risen in the mind of the public; and it is really up to Mr. Tennant to master the following simple definition: Enteric fever—better still, the enteric fevers—should be reserved to cover all diseases occasioned by organisms of the enteric group: that is to say, to cover typhoid fever, paratyphoid A fever, paratyphoid B fever, and any more varieties of these diseases which may hereafter be discovered. Typhoid fever should be reserved for that variety of enteric fever which is caused by *Bacillus typhosus*; paratyphoid A fever for that caused by *B. paratyphosus A*, and so on. The muddled phraseology employed in Parliament is directly responsible for much misunderstanding: either Mr. Tennant's informant on these questions is behind the march of scientific progress, or he himself does not "follow his brief" as closely as he ought to.

### THE LATEST ADDITION TO HOSPITAL HISTORIES.

Mr. Henry D. Roberts, director of the Public Library, Museum, and Fine Art Gallery, Brighton, has done a useful piece of work by writing a pamphlet describing the evolution of the Brighton Pavilion into a hospital for Indian troops. It is true that, from the point of view of the writer, the use of the Pavilion as a hospital is only the latest episode in the curious story which he unfolds; but, at any rate, we may be glad to have a no less curious chapter in hospital history thus preserved for us. From 1784, when the original farmhouse and the site were first altered to meet the whim of the Prince Regent, who desired a kind of "stately pleasure-dome" (with about as much architectural sense as is usually to be found in exhibition buildings), till to-day the Pavilion at Brighton has been used more and more to falsify its original function by becoming increasingly employed for public purposes. With its present features, as a hospital for Indians, we have dealt in previous issues; but the whole building deserves a brief history, and it receives its due in Mr. Roberts' interesting pamphlet.

### A MINISTRY OF SCIENCE.

In a discussion proceeding in the lay Press upon the relation of science to the State, accompanied by a plea for the endowment of research, it was argued that the best means of utilising inventive talent in the service of the State would be to form a Ministry of Science to "administer the progressive forces of modern knowledge." The attentive reader will not fail to note the common opinion that no suggested new department of importance can be regarded as ideally complete unless, or until, a seat in the Cabinet be given to its chief. We believe this view to be utterly fallacious, for Cabinet Ministers as a class have most of their interests outside their departments. State recognition and State aid may be desirable, and, indeed, a necessity, but these blessings may be obtained without a figurehead in the political game at Westminster. We do not possess a Ministry of Fine Arts; and have we any real reason to regret not having copied our friends the French in this respect? The moral is to develop our existing resources without bothering to create Cabinet offices for the new departments which grow up. Civilisation is said to be the creation of secondary needs by the provision of machinery for the satisfaction of primary ones; and science and art, like true pioneers, must be ahead of the State. The State's function is to give them opportunities by grants in aid and so on, but to incorporate them in Government departments is as likely to embarrass them as complete neglect would do.

### THE HOUSING OF MUNITION WORKERS.

One of the new problems for the Municipality and the medical officer of health that the war has created is the necessity for housing the workers in the new industries which have suddenly sprung up. That the problem is really a very large one in some districts can be seen from its dimensions at Sheffield, where the Estates Committee is faced with the need for providing for 5,000 munition workers, whose housing difficulties have been so great that the Lord Mayor has been compelled to appeal to the citizens to let apartments to them. This appeal, it is understood, has been successful, but the problem is not solved, and, therefore, the city architect has been busy preparing plans for temporary dwellings, to consist of hostels and colony blocks, while some beginning is being made with the erection of new permanent houses. When the time comes for the medical officer of health to write his report, there should be much interesting material on this head, especially as regards the temporary dwellings, for while there can be little doubt that the war has improved the design and quality of these for institutional purposes, the cheap, hygienic, and satisfactory dwelling-house for temporary purposes also offers a field for improvement, in the same way that this influx of munition workers brings to light a novel aspect of the housing question.

### THE MEDICAL EXAMINATION OF RECRUITS.

A good deal of discontent is manifest in various quarters over the medical examination of recruits, especially during the Derby boom. On the one hand, there are allegations that the medical inspections were so scamped that the majority of them will have to be done again; and that individual medical men have gained ten, or even more, pounds in a day by these scamped inspections at half-a-crown each. On the other hand are counter-assertions that two guineas a day is the maximum allowed by Government, and that some doctors were entirely cut off from their private practice by being called upon to examine recruits all day and half the night. Further, it would appear that many medical men who do recruiting work have no conception of the importance of their work and of the principles upon which it should be based; while there are many other men, some of them with military experience, who desire to get recruiting appointments, but cannot obtain any. Broadly speaking, many of these difficulties are inherent drawbacks of the voluntary system and of our lack of organisation and preparation for war as a nation. At the same time, it is probably true that much more might be done than is done at present to mitigate or avoid them, and that many of the complaints against both the War Office system and against individual members of the medical profession are well founded.

### THIS WEEK'S DRUG MARKET.

Business has been somewhat quieter during the past week, but there is a healthy undercurrent of inquiry. The scarcity of synthetic drugs is becoming more and more pronounced, and prices, in consequence, continue to tend in an upward direction. Atropine has now become so scarce that it is difficult to buy the smallest quantities even at extreme prices. Cocaine is rather dearer, and if the demand continues to be good, it is probable that there will be a further advance. Bromides are unchanged in value, but makers are not able to meet the demand promptly. On the other hand, the supply of iodides now seems to be adequate for all reasonable requirements. Eucalyptus oil is again dearer. The high price of ipecacuanha is firmly maintained. At the recent public sale of drugs in Mincing Lane large supplies were offered, the most noteworthy features being advances in the prices of senna and honey.

